# Supplementary material for: Unraveling NPR-like Family Genes in Fragaria spp. Facilitated to Identify Putative NPR1 and NPR3/4 Orthologues Participating in Strawberry-Colletotrichum fructicola Interaction
Source: Plants (Basel). 2022 Jun 16;11(12):1589. doi: 10.3390/plants11121589 (PMC9229442; doi:10.3390/plants11121589)
Supplement: Supplementary file 1 [file plants-11-01589-s001.zip › plants-1761067-supplementary figures.pdf]

Figure S1. Comparing motif compositions of NPR1-like deduced proteins from strawberry with Arabidopsis. Boxes in different colors represent distinct motifs identified at MEME (http://meme-suite.org, accessed on 11 Oct. 2021). The p-value shows the degree for overall matching to the con-sensus motif model.

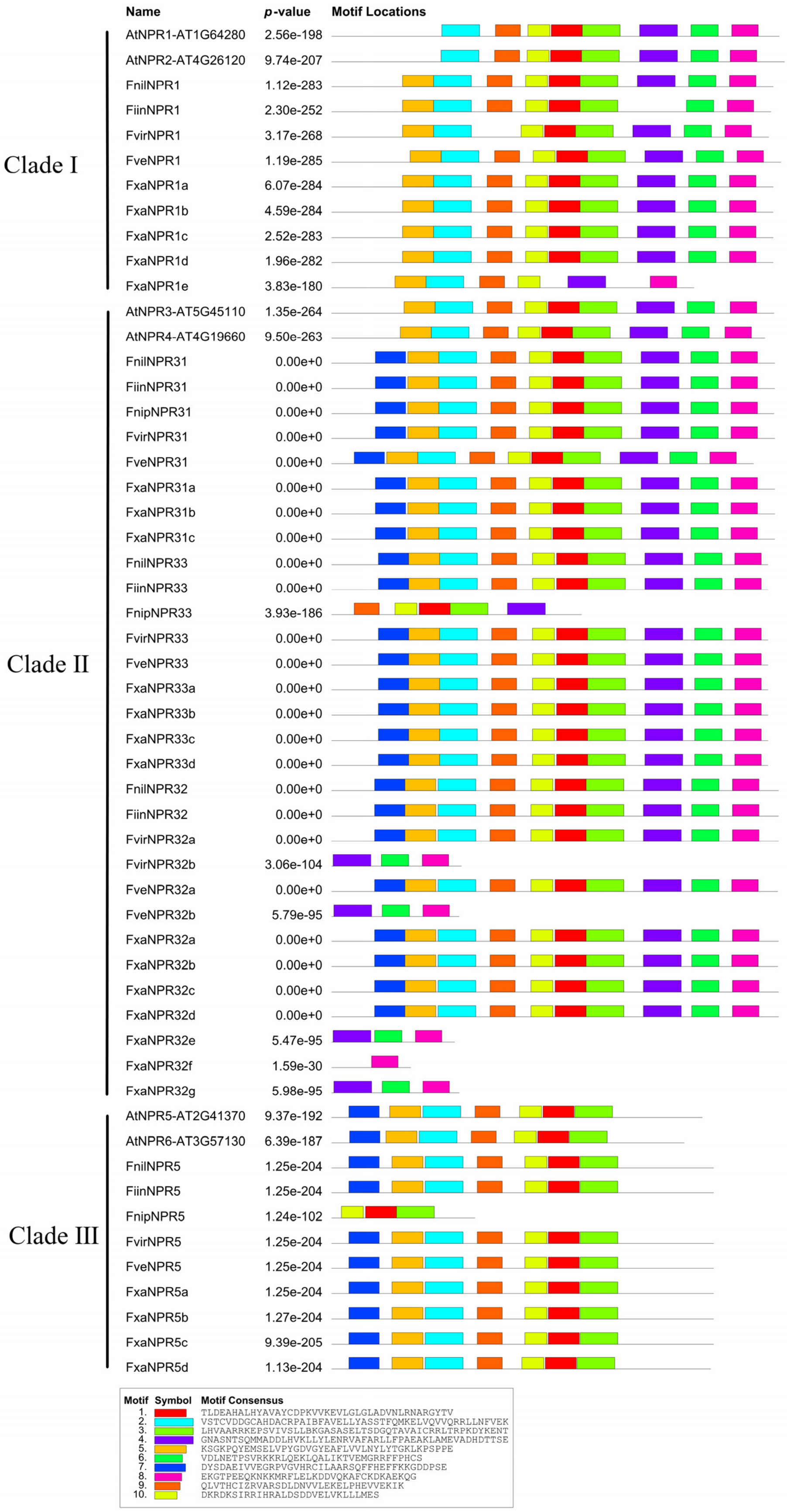

Figure S2. Sequence alignment for NPR1-like proteins from *Arabidopsis* and strawberry. (**A**) Alignment of six NPR1-like proteins from both *Fragaria vesca* and *Arabidopsis*. (**B**) Alignment of 11 amino acid sequences of clade I NPR proteins including AtNPR1-2, five from *F.* × *ananassa*, and one from *F. vesca*, *F. viridis*, *F. iinumae*, and *F. nilgerrensis*, respectively. (**C**) Alignment of 32 amino acid sequences of clade II NPR proteins including AtNPR3-4, 14 from *F.* × *ananassa*, and two from *F. nipponica*, three from both *F. iinumae* and *F. nilgerrensis*, four from both *F. vesca* and *F. viridis*. (**D**) Alignment of 11 amino acid sequences of clade III NPR proteins including AtNPR5-6, four from *F.* × *ananassa*, and one from *F. vesca*, *F. viridis*, *F. iinumae*, *F. nipponica*, and *F. nilgerrensis*, respectively. The conserved IkB-like phosphodegron, BTB/POZ domains, ankyrin repeats, putative hinge regions, NIMIN1/2-binding regions and nuclear localization signals (NLSs) are underlined. The six cysteine residues involved in oligomer formation are marked with asterisks. The third SUMO-interaction motif (SIM) consensus [VIL]-[VIL]-x-[VIL] is indicated in an ellipse, and the SA-binding core (SBC) at the C-terminal is framed.

A

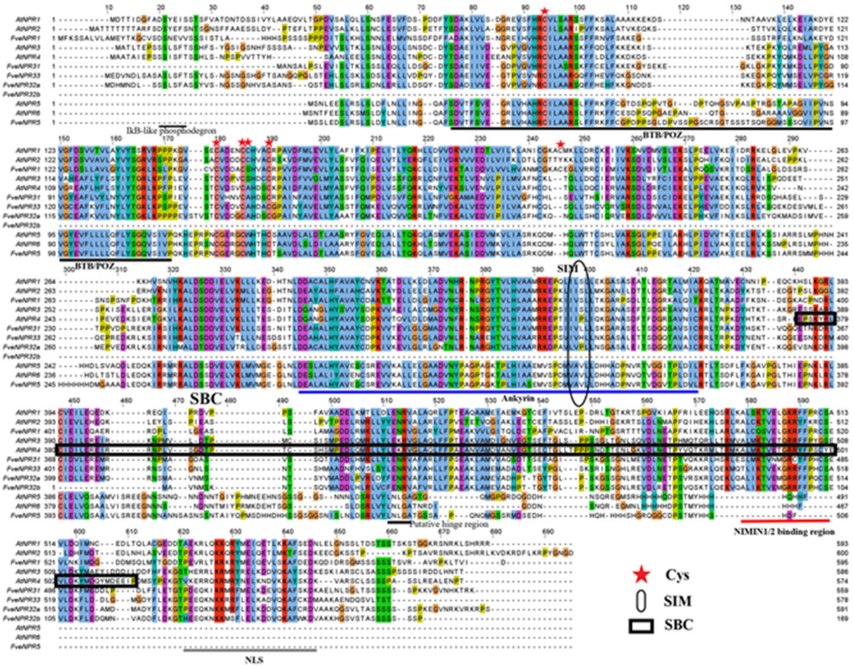

B

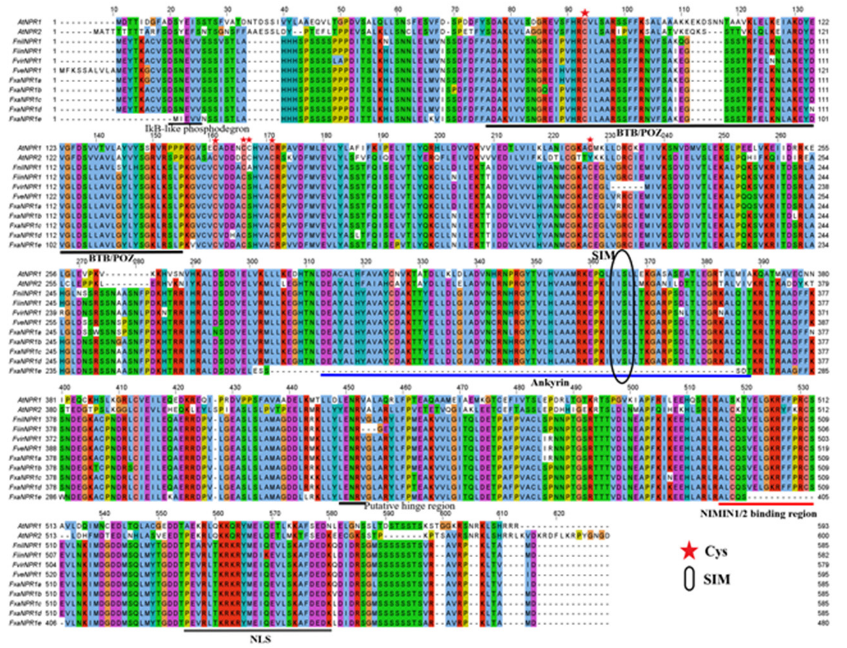

C

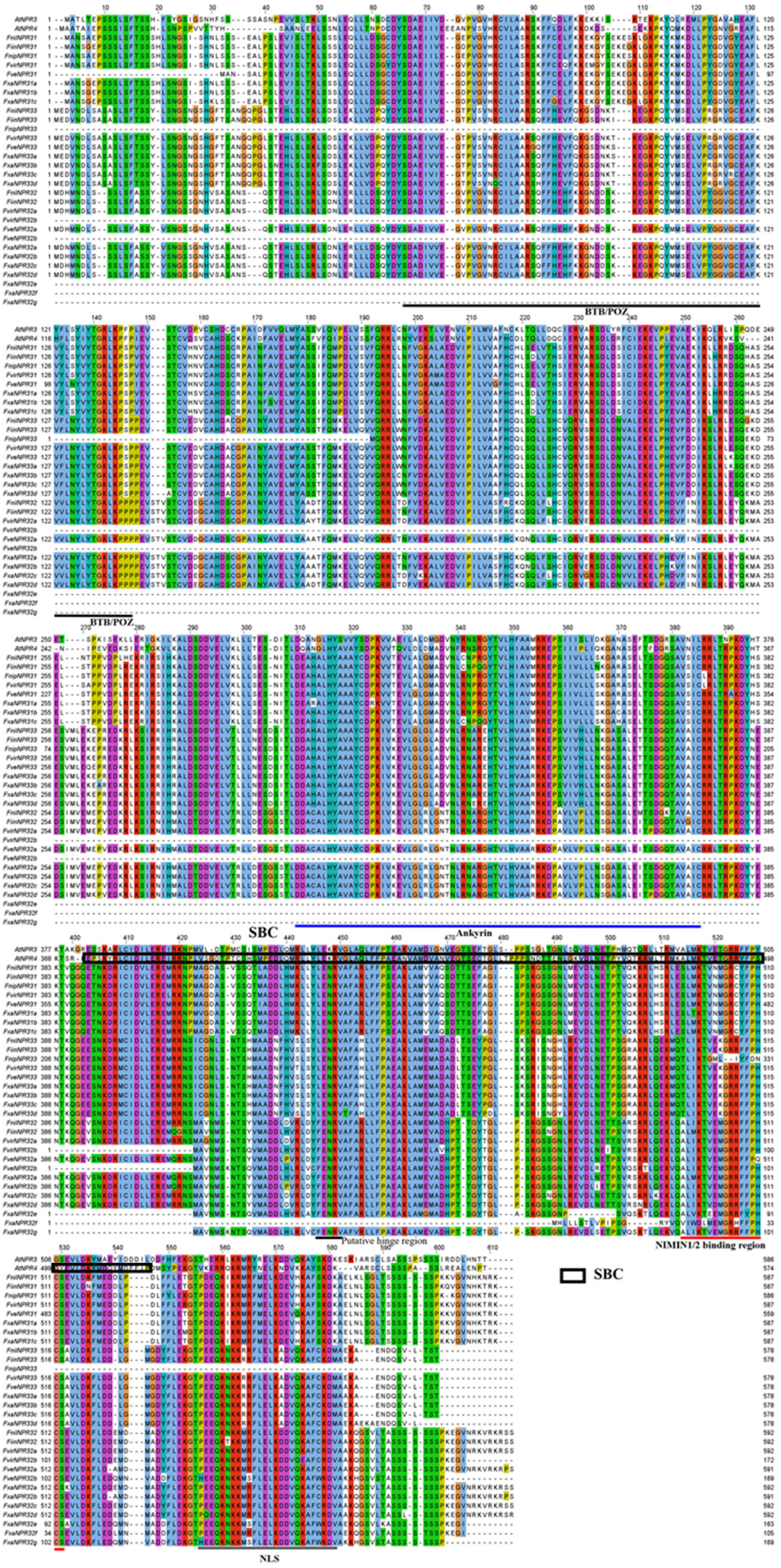
BTB/POZ
BTB/POZ
SBC
Ankyrin
Putative hinge region
NIMIN1/2 binding region
SBC
NLS

D

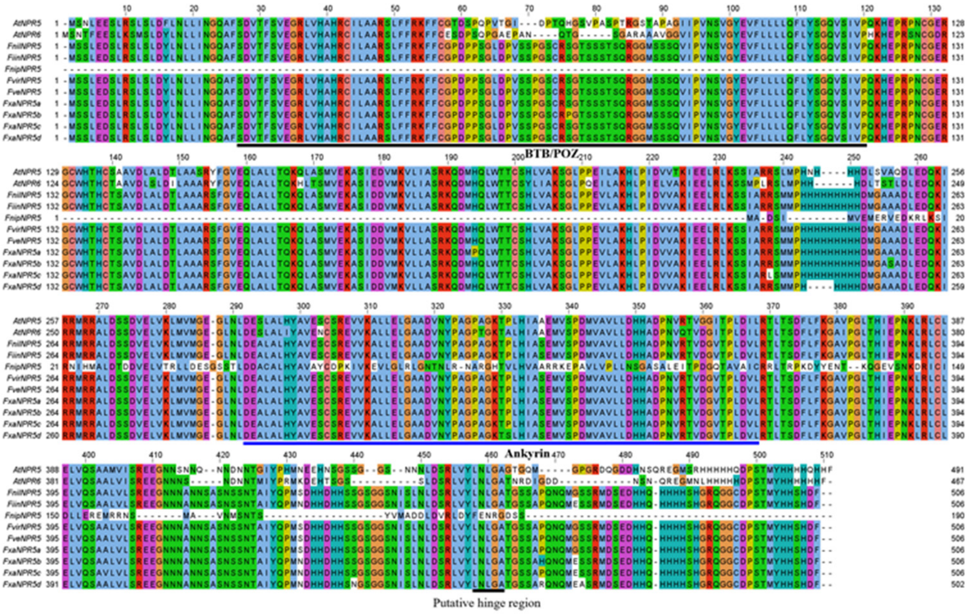

Figure S3. RNA-seq data showing the stable expression of genes coding proteins potentially directly or indirectly interacting with NPR1-like proteins in strawberry during *C. fructicola* invasion. The heatmap was generated using RPKM values normalized via Log_2-transformation in strawberry mock-treated or infected by *C. fructicola* at 24, 72, or 96 h post inoculation (hpi). RNAseq data was reported previously [35].

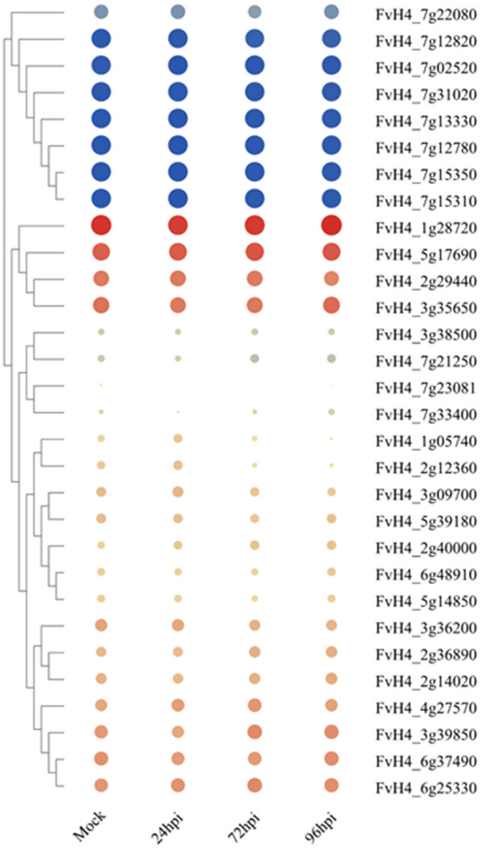

F. vesca V4.0a2	*F. vesca* V1.0-hybrid	NCBI Reference Sequence	Category	Annotation
FvH4_7g22080	gene26637	XP_004307539.1	UBE	ubiquitin-conjugating enzyme E2 7-like
FvH4_7g12820	gene18960	XP_004306863.1	Ring_U box	RING-box protein 1a-like
FvH4_7g02520	gene14201	XP_004308610.1	Ring_U box	RING-box protein 1a-like
FvH4_7g31020	gene13109	XP_004309160.1	SPCH	transcription factor SPEECHLESS-like
FvH4_7g13330	gene19132	XP_004306835.1	CSN	COP9 signalosome complex subunit 1-like
FvH4_7g12780	gene18964	XP_004306867.1	Ring_U box	RING-box protein 1a-like
FvH4_7g15350	gene19019	XP_004308337.1	WRKY22	WRKY transcription factor 22-like
FvH4_7g15310	gene19023	XP_004306735.1	ACS	1-aminocyclopropane-1-carboxylate synthase-like
FvH4_1g28720	gene25474	XP_004288382.1	EIN3	protein ETHYLENE INSENSITIVE 3-like
FvH4_5g17690	gene09341	XP_004299508.1	MAPK	mitogen-activated protein kinase kinase
FvH4_2g29440	gene11666	XP_004291469.1	HY5	transcription factor HY5-like
FvH4_3g35650	gene14141	XP_004294573.1	UBE	ubiquitin-conjugating enzyme E2 7-like
FvH4_3g38500	gene07891	XP_004294238.1	DDB2	DNA damage-binding protein 2-like
FvH4_7g21250	gene26729	XP_004308897.1	PTP	protein tyrosine phosphatase MKP1-like
FvH4_7g23081	gene26312	XP_004307466.1	BPM	BTB/POZ and MATH domain (adapter proteins)
FvH4_7g33400	gene12410	XP_004307881.1	CAND1	Cullin associated NEDD8 dissociated protein 1-like
FvH4_1g05740	gene11873	XP_004288882.1	PP2C	protein phosphatase 2C and cyclic nucleotide-binding kinase
FvH4_2g12360	gene27498	XP_004290440.1	PTP	protein tyrosine phosphatase PTP1-like
FvH4_3g09700	gene00121	XP_004304711.1	DDB1	DNA damage-binding protein 1-like
FvH4_5g39180	gene02282	XP_004300450.1	Vta1 like	a positive regulator of Vps4.
FvH4_2g40000	gene15122	XP_004291888.1	CSN	COP9 signalosome complex subunit 4-like
FvH4_6g48910	gene03848	XP_004304309.1	CUL3a	Cullin-RING ubiquitin E3 ligase
FvH4_5g14850	gene26020	XP_004299356.1	PTP	protein tyrosine phosphatase PTP1-like
FvH4_3g36200	gene14078	XP_004294513.1	APK1	protein kinase APK1A, chloroplastic-like
FvH4_2g36890	gene08701	XP_004291259.1	CSN	COP9 signalosome complex subunit 5a-like
FvH4_2g14020	gene32629	XP_004292529.1	VIP1	transcription factor VIP1-like
FvH4_4g27570	gene03759	XP_004299090.1	CSN	COP9 signalosome complex subunit 2-like
FvH4_3g39850	gene28174	XP_004294758.1	WRKY25	WRKY transcription factor 25-like
FvH4_6g37490	gene05705	XP_004303847.1	MAPK	mitogen-activated protein kinase kinase
FvH4_6g25330	gene21882	XP_004303596.1	HBP-1b	transcription factor HBP-1b(c38)-like
